# Recurrent Jejunal Melanoma Presenting as Obscure Gastrointestinal Bleeding: A Diagnostic Challenge

**DOI:** 10.14309/crj.0000000000002296

**Published:** 2026-09-17

**Authors:** Hamza Almusabeh, Daniel Teran, Dayyan Adoor, Ayowumi A. Adekolu, Abdullahi Adedotun Sulaiman, Shyam Thakkar

**Affiliations:** 1 Division of Internal Medicine, West Virginia University, Morgantown, WV; 2 Division of Gastroenterology and Hepatology, West Virginia University, Morgantown, WV; 3 Division of Pathology, West Virginia University, Morgantown, WV

**Keywords:** melanoma, gastrointestinal bleeding, jejunal mass, small bowel, double balloon enteroscopy

## Abstract

Melanoma commonly metastasizes to the gastrointestinal tract but is often asymptomatic. Symptomatic small intestinal metastatic melanoma presenting with overt gastrointestinal bleeding is uncommon and may be difficult to diagnose when the initial radiographic and endoscopic workup is unrevealing. We report a 47-year-old patient with prior stage III cutaneous melanoma status post wide local excision and sentinel lymph node biopsy, who declined adjuvant immunotherapy and surveillance, presenting with acute blood loss anemia occurring 3 years after definitive treatment. After nondiagnostic radiographic evaluation, esophagogastroduodenoscopy, colonoscopy, and push enteroscopy, anterograde double-balloon enteroscopy revealed an actively bleeding ulcerated midjejunal mass, later measuring 6 cm on surgical pathology, with pathology confirming the diagnosis of metastatic melanoma.

## INTRODUCTION

Melanoma is one of the malignancies most likely to metastasize to the gastrointestinal tract (GIT).^1^ Autopsy data of patients with melanoma suggest that up to 43.5% of patients harbor metastatic GIT lesions.^2^ Although symptomatic GIT metastasis may occur at the time of primary diagnosis, they often present several years later. Common presentations include nonspecific symptoms such as abdominal pain, but they may rarely present as bowel obstruction, perforation, or gastrointestinal bleeding.^3^ Gastrointestinal involvement is a poor prognostic feature of melanoma.^3^ We report a case of recurrent melanoma presenting with gastrointestinal bleeding occurring 3 years after initial definitive treatment.

## CASE REPORT

A 47-year-old man with a history of end-stage renal disease and right neck staged pT2bN1a status post wide local excision and sentinel lymph node biopsy with no documented evidence of recurrence for 3 years presented to the emergency department with progressively worsening fatigue and melena. Patient's original melanoma had a Breslow depth of 1.5 mm, ulceration, and 2 mitoses/mm^2^. Wide local excision showed no residual melanoma, negative resection margins, no lymphovascular invasion, and 1 of 3 sentinel lymph nodes positive without extranodal extension. Gross primary tumor size was not reported in the available pathology record. Baseline positron emission tomography-computed tomography (PET/CT) after resection showed no definite metastatic disease. Adjuvant pembrolizumab for 1 year and nodal-basin ultrasound surveillance every 3–4 months were recommended; however, the patient declined adjuvant therapy and surveillance.

Initial laboratory workup at an outside facility was notable for a hemoglobin of 5.3 g/dL. Before transfer, he reported 6–7 weeks of melena and fatigue and required approximately 2 units of packed red blood cells weekly for 5 weeks, with 12 units transfused during the outside hospitalization. Outside workup included esophagogastroduodenoscopy showing gastritis without active bleeding, colonoscopy showing hemorrhoids without active bleeding and melanic stool throughout the colon with no active source, CT abdomen/pelvis with contrast showing no acute gastrointestinal bleeding, nuclear medicine bleeding scan concerning for bleeding in the left abdomen, mesenteric angiography without active extravasation or pseudoaneurysm, push enteroscopy showing hemorrhagic gastritis without significant duodenal or proximal jejunal pathology, and video capsule endoscopy showing small-bowel erosions vs arteriovenous malformations with oozing beyond the reach of standard endoscopy. Bone marrow biopsy showed no evidence of acute leukemia or non-Hodgkin lymphoma.

On transfer and admission to our facility, laboratory evaluation showed severe normocytic, normochromic anemia, with a hemoglobin of 7.2 g/dL and mean corpuscular volume of 87.4 fL. Peripheral blood smear demonstrated anisopoikilocytosis with occasional ovalocytes and basophilic stippling without schistocytes. The following day, hemoglobin dropped to 6.5 g/dL. Platelets, white blood cell count, and coagulation studies were within normal limits. Overall, the presentation was most consistent with acute blood loss anemia in the setting of overt gastrointestinal bleeding.

**Figure 1.**
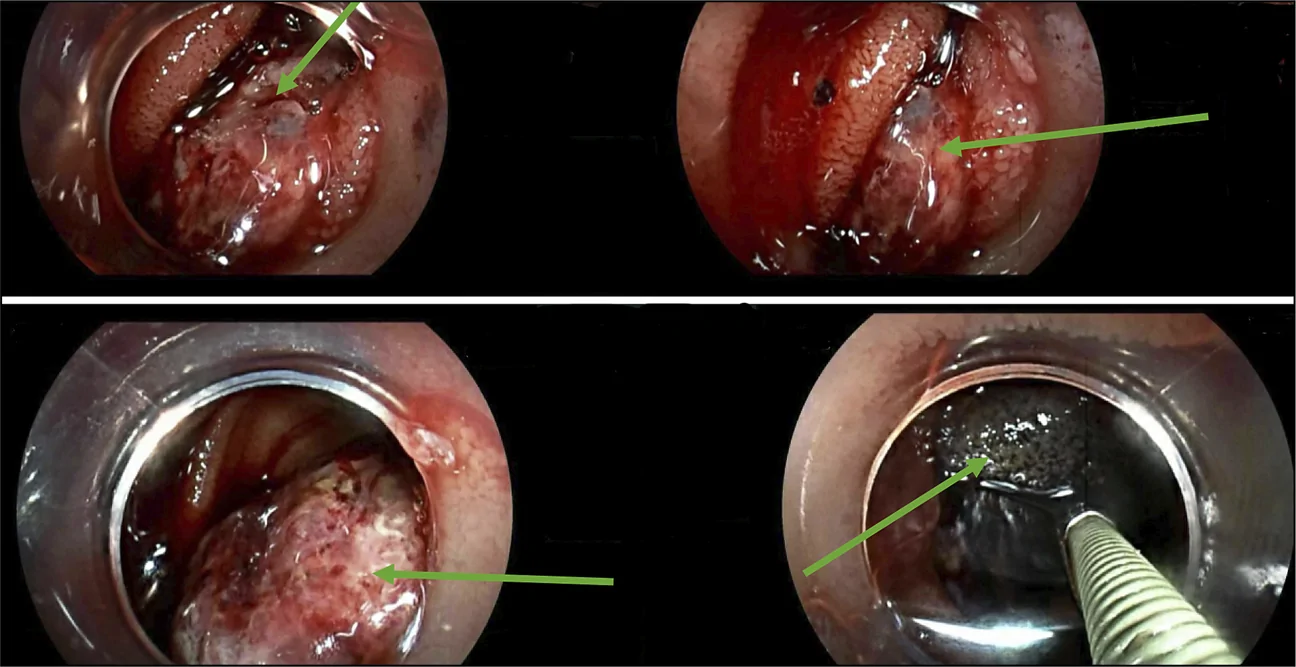
Bleeding ulcerated fungating jejunal mass. Arrows indicate the exact pathology (ulcerated jejunal mass).

**Figure 2.**
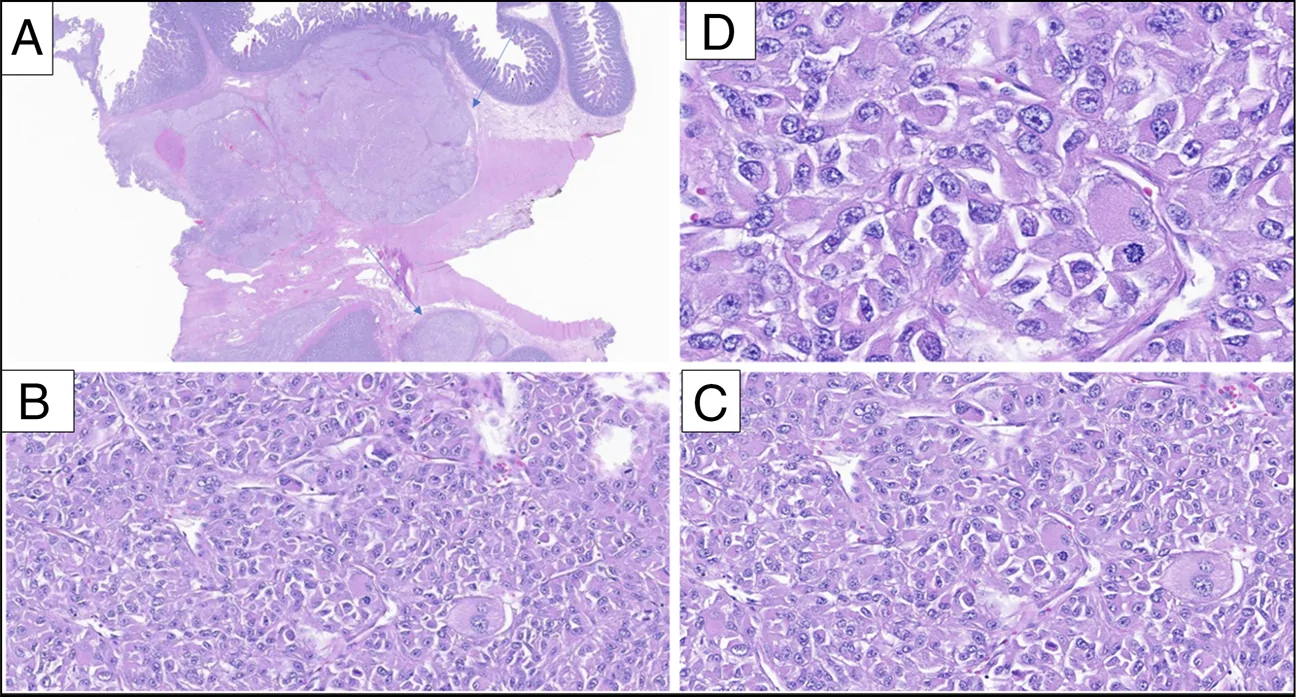
(A) Low-power view showing a nodular submucosal mass involving the muscularis propria (blue arrow) (original magnification 2×). (B)–(D) Cytology highlights cells with eosinophilic cytoplasm, nuclear enlargement and variability, prominent nucleoli, and atypical mitoses (original magnification 20× in [B] and [C], 40× in D).

**Figure 3.**
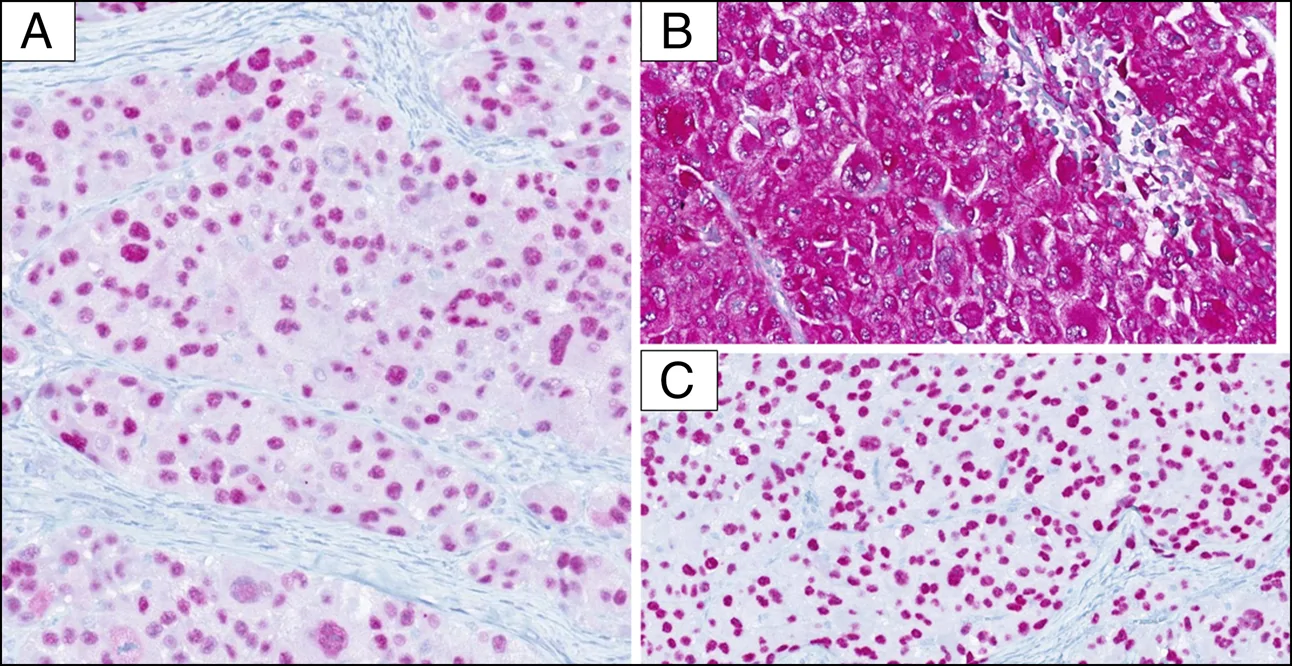
Panels (A)–(C): Immunohistochemical stains highlight diffuse nuclear PRAME positivity (A), strong S100 reactivity (B), and nuclear SOX10 expression (C) (original magnification 40×). The combined immunoprofile is diagnostic of metastatic malignant melanoma.

Given persistent anemia and concerns for a small bowel bleeding source, anterograde double balloon enteroscopy (DBE) was performed and revealed a large fungating, actively bleeding, and ulcerated mass in the midjejunum (Figure 1). Biopsies were obtained, and temporary hemostasis was achieved using hemostatic spray. The lesion was tattooed with 2 mL of India ink, and no bleeding was present at the end of the procedure. The patient subsequently underwent laparoscopic-assisted jejunal small-bowel resection with primary side-to-side functional end-to-end stapled anastomosis for definitive management of the bleeding lesion. Surgery revealed no ascites or visible peritoneal/gross metastatic disease. The tattooed jejunal segment was identified, and pathologic examination of the mass confirmed metastatic melanoma (Figures 2 and 3). The resected specimen consisted of a 16.0 × 4.0 cm segment of small bowel with 2.5 cm of attached mesentery and a 6.0 × 4.0 cm exophytic polypoid mass. The mass was 2.5 cm from the distal margin and 5.0 cm from the proximal margin, extended 3.0 cm deep, and grossly involved the mucosa, submucosa, muscularis, and appeared to involve the serosa. Resection margins were negative, and no lymph nodes were identified despite complete mesenteric submission. Immunohistochemistry showed tumor cells positive for S100, SOX10, and PRAME, with focal HMB45 and MART1 positivity, supporting metastatic melanoma. Postoperatively, he had no immediate complications and was discharged on postoperative day 1. Available records after discharge were limited due to the patient being lost to follow-up.

## DISCUSSION

Symptomatic gastrointestinal metastatic melanoma is rare and may be difficult to diagnose because of the nonspecific presentation.^3–5^ A study reported that gastrointestinal melanoma (GIM) metastases are observed in up to 60% of patient autopsies, whereas clinical antemortem detection can be as low as 1%–5%.^6^ A UK-based study reported only a 17% incidence of gastrointestinal bleeding as a presenting symptom among those diagnosed with GIM.^7^ The challenge is further compounded by the limited sensitivity of conventional CT scans, which detect only 60%–70% of metastatic cases.^8^ In comparison, one study found that 18F-fluorodeoxyglucose PET/CT had 85.7% sensitivity for detecting GIM metastases, which may improve detection compared with CT alone.^9^

In our patient, CT angiography did not detect any suspicious lesions and initial endoscopic workup, including an esophagogastroduodenoscopy, colonoscopy, and push enteroscopy was negative. The culprit metastatic lesion was ultimately discovered after small-bowel evaluation through video capsule endoscopy and anterograde DBE. Despite advances in diagnostic modalities, similar cases with delayed or difficult diagnosis have been described in the literature.^9–12^

Another important consideration when evaluating metastatic small-bowel melanoma is its potential for late, nonprimary site recurrence after index definitive treatment of the primary The median interval between treatment of the first melanoma and diagnosis of gastrointestinal metastasis is 48 months but has ranged from 0 months to 40 years, with some reports exceeding this period.^10,13^ It has also been reported in the literature that intestinal melanoma could be the first sign of recurrence.^14,15^ In our case, the diagnosis was established almost 3 years after the definitive treatment. This occurred after the patient declined recommended adjuvant pembrolizumab and surveillance following node-positive cutaneous melanoma. This emphasizes that metastatic melanoma must remain in the differential diagnosis for patients presenting with gastrointestinal symptoms.

Management of midgut metastatic melanoma is guided by symptoms and overall disease burden. The reported 5-year survival rate is approximately 22.5%.^16^ Treatment options include surgical resection, systemic therapies, or a combination of both.^17–19^ Surgical resection is often used for symptomatic small-bowel metastases causing bleeding, obstruction, perforation, or intussusception, ideally in combination with multidisciplinary evaluation for perioperative or postoperative immunotherapy when clinically appropriate.^16–19^ In our patient, surgical resection was performed for bleeding control. Systemic therapy was not given before surgery because of active bleeding and transfusion dependence. Postoperatively, PET/CT staging and oncology follow-up were planned, but subsequent treatment records were limited by poor follow-up.

This case highlights the difficulty in diagnosing small-bowel metastatic melanoma when it presents as overt obscure gastrointestinal bleeding. In patients with a history of melanoma, metastatic disease should remain in the differential diagnosis even years after definitive treatment. When standard endoscopic and radiologic evaluation is unrevealing, persistent overt obscure gastrointestinal bleeding should prompt consideration of a small-bowel source and further evaluation with video capsule endoscopy and DBE. A limitation of this report is the lack of longer-term follow-up after jejunal resection, which limits assessment of prognosis, recurrence, systemic therapy use, and disease control.

## DISCLOSURES

Author contributions: H. Almusabeh and D. Teran contributed to literature review and manuscript drafting. AA Adekolu and D. Adoor contributed to endoscopic image acquisition, interpretation, and manuscript review. AA Sulaiman contributed to interpretation of pathologic images. S. Thakkar contributed to critical revision of the manuscript. All authors approved the final manuscript. H. Almusabeh is the article guarantor.

Financial disclosure: None to report.

Informed consent was obtained for this case report.

## ABBREVIATIONS:

DBE: double ballon enteroscopy
GIM: Gastrointestinal melanoma
GIT: Gastrointestinal tract
PET/CT: Positron emission tomography- computed tomography
VCE: Video capsule endoscopy
